# Prevalence and determinants of self-medication practice among selected households in Addis Ababa community

**DOI:** 10.1371/journal.pone.0194122

**Published:** 2018-03-26

**Authors:** Mensur Shafie, Mebrahtu Eyasu, Kedija Muzeyin, Yoseph Worku, Sagrario Martín-Aragón

**Affiliations:** 1 Saint Paul’s Hospital Millennium Medical College, Department of Pharmacology, Addis Ababa, Ethiopia; 2 Saint Paul’s Hospital Millennium Medical College, Department of Nursing, Addis Ababa, Ethiopia; 3 Saint Paul’s Hospital Millennium Medical College, Department of Public Health, Addis Ababa, Ethiopia; 4 Complutense University of Madrid, School of Pharmacy, Department of Pharmacology, Madrid, Spain; Kasturba Medical College Mangalore, INDIA

## Abstract

**Background and aim:**

Self-medication (SM) is one part of self-care which is known to contribute to primary health care. If practiced appropriately, it has major benefits for the consumers such as self-reliance and decreased expense. However, inappropriate practice can have potential dangers such as incorrect self-diagnosis, dangerous drug-drug interactions, incorrect manner of administration, incorrect dosage, incorrect choice of therapy, masking of a severe disease, and/or risk of dependence and abuse. The main objective of this study was to assess the prevalence and determinants of the self-medication practice (SMP) in Addis Ababa.

**Methodology:**

A community based cross-sectional study was conducted among selected households in Addis Ababa from April 2016 to May 2016, with a recall period of two months before its conduction. Trained data collectors were employed to collect the data from the 604 sampled participants using pre-tested and validated questionnaires.

**Result:**

Among the 604 participants involved in this study, 422 (69.9%) were female and 182 (30.1%) were male and there was a mean age of 41.04 (± 13.45) years. The prevalence of SM in this study was 75.5%. The three most frequently reported ailments were headache 117 (25.7%), abdominal pain 59 (12.9%) and cough 54 (11.8%). The two main reasons for SM were mildness of illness 216 (47.4%) and previous knowledge about the drug 106 (23.2%). The two most frequently consumed medications were paracetamol 92 (20.2%) and traditional remedies 73 (16.0%), while drug retail outlets 319 (83.3%) were the main source of drugs. The two most frequently reported source of drug information were health professionals 174 (45.4%) and experience from previous treatment 82 (21.4%). Moreover, there were statistically significant differences among respondents who reported practicing SM based on income and knowledge about appropriate SMP.

**Conclusion and recommendation:**

Self-medication was practiced with a range of drugs from the conventional paracetamol and NSAIDs to antimicrobials. Being that the practice of SM is inevitable, health authorities and professionals are highly demanded to educate the public not only on the advantages and disadvantages of SM but on its proper use.

## Introduction

According to target eight of the Millennium Development Goal (MDG), essential drugs should be accessible and affordable in developing countries with the appropriate information and communication [1]. In line with this, self-medication (SM) is one of the contributors for rational drug use. SM is identified as one of the key aspects of primary health care by the World Health Organization (WHO) [2] and World Self-Medication Industry [3]. SM is one aspect of self-care which people practice for themselves in order to maintain health or prevent and deal with illnesses [4]. SM is defined as the selection and use of medicinal products by the consumer to treat self-recognized illnesses or its symptoms, or the intermittent or continued use of a medication prescribed by a physician for a chronic or recurring disease or symptom [2].

The prevalence of SM has sharply increased throughout the world. There are reports showing that up to 80% of all drugs are purchased without any prescription in developing countries, [5] which is substantiated by reports that [6,7,8] the prevalence of self-medication in developing countries is in the range of 12.7% to 95%. In addition, the few studies performed in Ethiopia reported a high prevalence of SM. Prevalence ranging from 27.6% in the Jimma town [9] to 38.5% among students from the Gondar University [10] to 39% in the Assendabo town [4] have been reported.

SM is one of the key contributors for making essential drugs accessible and affordable in a developing country [1]. One of the strategies to prevent diseases such as HIV/AIDs, and malaria is self-administration of medications for prevention. It is a vastly spreading concept advocated by various groups throughout the world including the WHO. If SM is practiced appropriately, SM is a major contributor to the health care system and can be beneficial in various aspects. Convenience, economical advantage, direct and rapid access to treatment, self-reliance in preventing or relieving minor symptoms, and improving a person’s active role in his/her own health care are some of the well-established advantages [3]. However, if not used as intended, it might pose serious problems such as drug toxicity, wasted medical resources, drug interactions, microbial resistance, drug dependence, and/or addiction [11].

In Ethiopia, and particularly in the selected households among the Addis Ababa community, the magnitude and pattern of self-medication practice (SMP), factors affecting SM, whether the practice is appropriate or not, whether the public is knowledgeable on the rational use and risk of the medicines, the common types of illnesses leading to SMP, and major types of drugs consumed have not yet been adequately studied. Therefore, the major aim of this study was to determine the prevalence and determinants of SM in selected households among the Addis Ababa community.

## Methodology

### Study area and period

The current study was conducted on selected households in Addis Ababa from April, 2016 to May, 2016. Addis Ababa is the capital city of the Federal Democratic Republic of Ethiopia and unlike to other regions of Ethiopia, it holds the highest concentration of health care facilities and health professionals. Nurses comprise the highest number among the estimated 10,000 health professionals that cater to the health-related needs of the city’s 3.3 million people. The city is divided in 12 sub-cities and 117 woredas. There are 12 public hospitals in the city: Tikur Anbessa Hospital, St. Paul’s Hospital, Yekatit 12 Hospital, Gandhi Memorial Hospital, Ras Desta Hospital, Zewditu Hospital, Menilik Hospital, St. Peter’s Hospital, Tirunesh-Beijing Hospital, Alert Center, Federal Police Referral and Army Hospitals. In addition, there are 31 private hospitals as well as many other health facilities. The health system in the city, which is also considered as the capital of Africa, is a two-tier system: hospitals and health centers [12].

#### Study design

A cross-sectional community-based survey was conducted by using semi-structured questionnaires.

### Source and study population

All households in Addis Ababa were the source population. Of these, selected households during the data collection period were used as the study population based on the sampling technique employed.

### Inclusion and exclusion criteria

#### Inclusion criteria

- Being a resident, preferably the adult household head, of Addis Ababa.

#### Exclusion criteria

- Those who were under 18 years of age, mentally incompetent, unable to listen or speak Amharic well and those who were unwilling were not included in the study.

### Sample size and sampling technique

#### Sample size

The sample size was calculated using the single population proportion formula and the 39% prevalence reported in Assendabo town [4].

$$n=\frac{{\left(Z\alpha |2\right)}^{2}\times p(1-p)}{d^{2}}*\mathrm{de}$$

Where: n is the sample size; Zα/2 is the standardized normal distribution value at the 95% confidence interval level, which is 1.96; p is the proportion of self-medication, 39%; d is the margin of error taken as 5%; de is the design effect for using multi-stage sampling taken as 1.5.

$$n=\frac{{\left(1.96\right)}^{2}\times 0.39(1-0.39)}{{\left(0.05\right)}^{2}}*1.5$$

n=549

Therefore, the final minimum sample size was 604 households with 10% considered contingency.

#### Sampling technique

For this study, a multistage sampling technique was used. Three Subcities of Addis Ababa (Gullele, Arada and Ldeta) were selected by lottery method and the sample size was apportioned based on the number of the households in each Subcity (Table 1). Woredas were selected from the Subcities using simple random sampling. Then, the sample allocated for each woreda was apportioned to the Woredas in the Subcity depending on the number of households in each woreda. The households in the woreda to be included in this study were selected by simple random sampling. The house number of the households within the woredas was used as a sampling frame. We selected each household through a random computer method. If one household was unwilling to participate, the next household would be taken.

**Table 1:** Allocation of the study participants from each of the selected woredas.

Sr. No.	Subcity	Woreda	Total number of households in the woreda	Sample obtained
1	Gulele	9	6470	68
		8	6349	66
		3	5256	55
		10	4366	46
2	Arada	6	5810	64
		7	5020	55
		10	3123	35
		1	2950	33
3	Ldeta	5	5874	56
		4	5328	51
		1	4500	43
		7	3314	32

### Data collection procedure

Ten data collectors with great communication skills in local languages were recruited. Training was given on how to hold an effective interview with the study units using the questionnaire. A structured questionnaire was employed to gather the necessary information on socio-demographic characteristics, prevalence, and determinants of SMP.

### Variables

#### Independent variables

- Age, Marital status, Ethnicity, Religion, Educational status, Income, Occupation, Family status, Condition of drug consumer, Knowledge about appropriate SMP, Types of drugs used for SM and diseases that lead to SM

#### Dependent variables

- Self-medication practice (yes or no)

### Operational definition

**Traditional medicine.** The use of different types of traditional medicine practices such as using plant materials, animal products and/or religious practices (use of holy water for drinking and washing, use of soil or mud from a religious site, etc.).

**Self-medication.** The self-reported treatment of common health problems by the study participants with modern and/or traditional medicines without direct medical or traditional healer supervision or intervention (further it might include consultation with pharmacists and extensive use of previous prescription drugs in the last two months).

**Knowledge on appropriate SMP.** Respondent was considered knowledgeable if he/she answered 50% or more of the questions associated with knowledge about appropriate SM correctly.

**SMP.** Action by which a study participant suspects his/her illness and has taken a drug (modern or traditional) in the past two months without prescription from a physician.

#### Physician

Any person who is medically qualified to prescribe medications.

### Data analysis

Data were cleaned, coded, and entered into the SPSS statistical package, latest version 21. Descriptive statistics was computed for the study variables. Frequency distribution tables were used to describe the findings and graphs were plotted. Association between dependent and independent variables was analyzed using odds ratio and chi-square tests. Association of variables was significant if the p-value was less than or equal to 0.05.

### Data quality control

The data collection tool was translated to Amharic and pre-tested for its accuracy, completeness, and consistency prior to actual collection on five percent of the sample population at two woredas, in Kolfe Keranio Subcity. Furthermore, the coordinator and principal investigator observed and gave feedback to the data collectors on a daily basis. Completeness, accuracy and clarity of the collected data were checked carefully by the principal investigators and any error was addressed on the following day before starting next day activities.

### Ethical clearance

The proposal was reviewed for any ethical issues by Saint Paul’s Hospital Millennium Medical College Internal Review Board. Participants’ information like name and address were not recorded and coding was used in the questionnaires. All data were kept confidentially and only used for the purpose of the current research. Written consent was obtained from each participant and all were clearly informed that they had the right to not participate if they were unwilling.

## Results

### Socio-demographic characteristics of study participants

A total of 604 study participants were involved in this study. Of the total, 235 (38.9%) were from Gulele subcity (68 from woreda nine, 66 from woreda five, 55 from woreda three and 46 from woreda ten), 187 (31.0%) were from Arada subcity (64 from woreda six, 55 from woreda seven, 35 from woreda ten and 33 from woreda one), and the rest, 182 (30.1%) were from Ldeta subcity (56 from woreda five, 51 from woreda four, 43 from woreda one and 32 from woreda seven).

The different socio-demographic characteristics of study participants are presented in Table 2. Most of the study participants (69.9%) were female. One hundred and eighty two (30.1%) of the study participants were in the age group of 35–44 and the mean (±SD) age of respondents was 41.04 (±13.45). The minimum and maximum age of study participants were 18 and 84, respectively (the range was 66). Three hundred and fifty eight (59.3%) of study participants were followers of Orthodox Christianity, (26.7%) were Muslim and (11.9%) were Protestant Christians. Two hundred and twenty one (36.6%) of study participants were Amhara followed by Oromo (26.5%) and Gurage (15.7%) in their ethnicity. Most of the study participants (67.5%) were married. The mean (±SD) monthly income of study participants was 1654.36 (±1284.94) birr. Two hundred and forty five (40.6%) of the study participants earned more than 1500 birr in a month. Two hundred and three (33.6%) of study participants had attended higher education, followed by secondary education (27.8%) and being able to read and write but having no formal education (24.7%). The occupation of two hundred and thirty one (38.2%) of study participants was employment in private organization followed by government employee and being a house-wife (25.8% and 16.2%, respectively). The household responsibility of majority of study participants was being mother (56.8%) followed by being a father (23.7%).

**Table 2:** Socio-demographic characteristics of study participants at Addis Ababa from April, 2016 to May, 2016.

Socio-demographic variable	Frequency	Percent
**Subcity**		
Gulele	235	38.9
Arada	187	31.0
Ldeta	182	30.1
**Sex**		
Female	422	69.9
Male	182	30.1
**Marital status**		
Married	408	67.5
Single	109	18.0
Separated	44	7.3
Divorced	43	7.1
**Age**		
18–24	32	5.3
25–34	182	30.1
35–44	157	26.0
45–54	131	21.7
≥ 55	102	16.9
**Religion**		
Orthodox Christian	358	59.3
Muslim	161	26.7
Protestant Christian	72	11.9
Others*	13	2.2
**Ethnicity**		
Amhara	221	36.6
Oromo	160	26.5
Gurage	95	15.7
Tigray	78	12.9
Silte	28	4.6
Others**	22	3.6
**Academic status**		
Illiterate	72	11.9
Read and write but no formal education	149	24.7
Primary education	12	2.0
Secondary education	168	27.8
Higher education	203	33.6
**House hold responsibility**		
Mother	343	56.8
Father	143	23.7
Child	82	13.6
Relative	36	6.0
**Occupation**		
Private employee	231	38.2
Government employee	156	25.8
House wife	98	16.2
Private owned job	56	9.3
Student	25	4.1
Unemployed	25	4.1
Pension	13	2.2
**Monthly income (birr)**		
< 500	135	22.4
500–1000	139	23.0
1001–1500	85	14.1
> 1500	245	40.6

*Catholic, Johova’s witness

**Hadya, Hadere, Kambata, Sidama,Wolayta

### Self-medication practice

**Fig 1:**
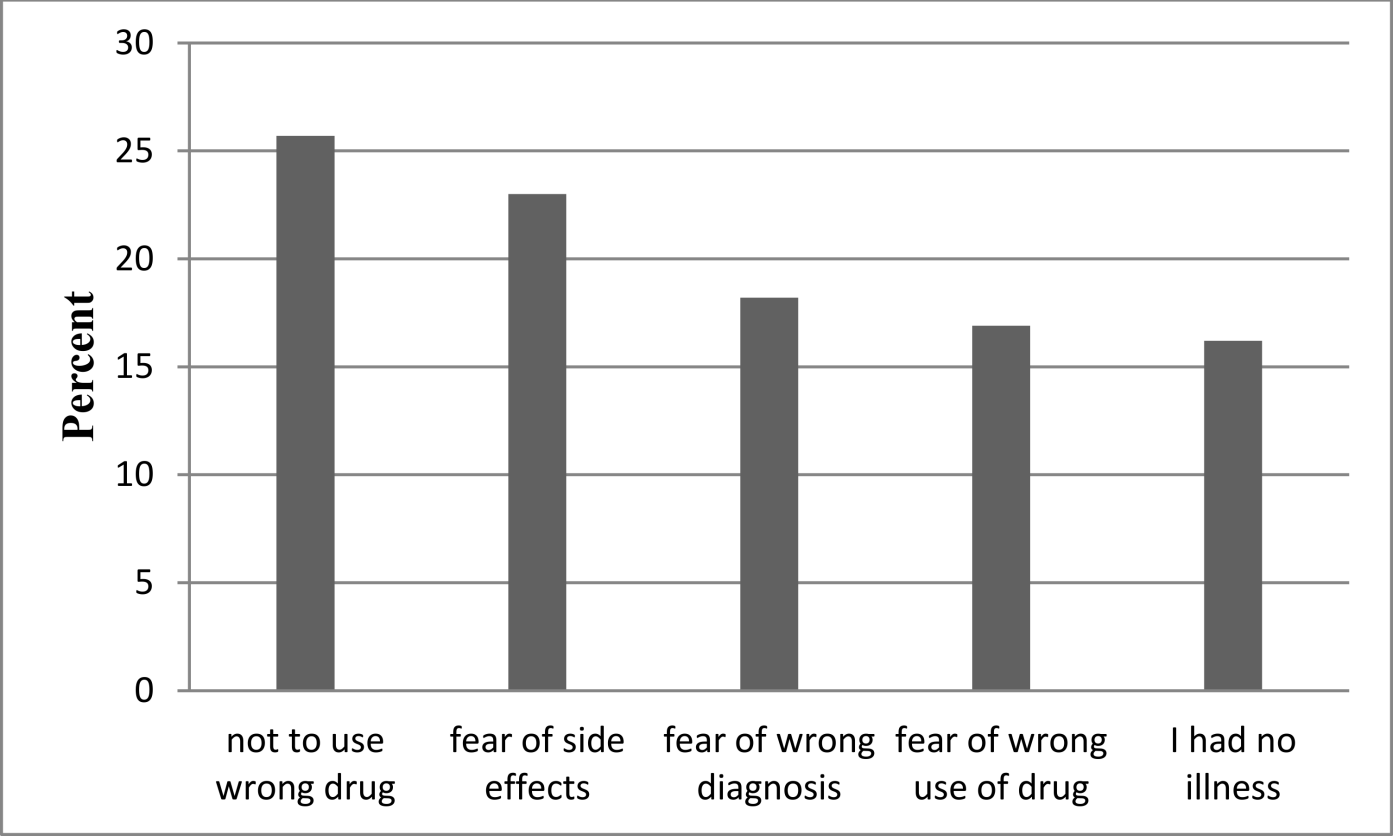
Study participant reasons for not practicing SM in Addis Ababa, from April, 2016 to May, 2016.

The finding of this study revealed that from the total 604 study participants, 456 (75.5%) practiced SM. The remaining 148 (24.5%) did not practice SM because of the following reasons: fear of using a wrong drug (25.7%), fear of side effects of drugs (23.0%), fear of wrong diagnosis of their illness (18.2%), fear of wrong use of drugs (16.9%), and absence of any ailment in the past two months (16.2%) **[**Fig 1**].**

Out of those respondents who practiced SM, the majority (66.9%) had used modern medicine followed by both modern and traditional medicine (17.1%) and traditional medicine (16.0%).

The source of information for those study participants who are users of modern medicine for SM is presented in Table 3. Advice from a health professional but without prescription covered 45.4%. Other sources of information included experience of previous treatment (21.4%), a friend’s advice (16.4%), self-decision (12.5%), and reading information in books or on the internet (4.2%).

**Table 3:** Practices related to SM in Addis Ababa residents from April, 2016 to May, 2016.

Variable	Frequency	Percent
**Source of information about modern medicine**		
Health professional but without prescription	174	45.4
Experience from previous treatment	82	21.4
Friend	63	16.4
Self-decision	48	12.5
Book/internet	16	4.2
**Source of modern medicines**		
Pharmacy/drug store	319	83.3
Neighbour	26	6.8
Remnant from previous treatment	25	6.5
Others*	13	3.4
**Way of requesting medicines from pharmacy/drug store**		
Mentioning the name of the drug	175	54.9
Telling symptoms of disease to a pharmacy professional	84	26.3
A piece of paper with the name of the drug	36	11.3
Taking the drug container	24	7.5
**Type of illness that become reason for self- medication**		
Headache	117	25.7
Abdominal pain	59	12.9
Cough	54	11.8
Diarrhea	43	9.4
Toothache	39	8.6
Stomach ache (dyspepsia)	29	6.4
Hypertension	13	2.9
Fever	11	2.4
Eye disease	11	2.4
Constipation	9	2.0
Asthma	5	1.1
Combination of illnesses**	27	5.9
Others***	39	8.9

*Friend, shop, super market and health professional but by the request of respondent

**Fever and cough, cough and headache, headache and abdominal pain, urinary tract infection and pain, cough and joint pain, toothache and abdominal pain, disease of thyroid gland and diarrhea, headache and dyspepsia, headache and eye disease, headache and hypertension, diarrhea and headache

***Common cold, *diabetes mellitus*, urinary tract infection, sore throat, tonsillitis, joint disease, eye disease, malaria, sinusitis, dysmenorrhea, ‘mich’, skin disease, heart disease, thyphoid.

This study has also assessed the kind of source of modern medicine in case of those respondents who used modern medicine for SM. The finding indicated that the source was drug retail outlets (pharmacy and drug store) in the majority (83.3%) of the cases. Other sources which constituted 6.8, 6.5, and 3.4%, respectively, included from a neighbor, remnants from previous treatment and other sources like a friend, supermarket, a shop, and from a health professional (Table 3).

Regarding the way of request of drug in case of those who obtained drugs from drug retail outlets, the majority (54.9%) pointed out mentioning the name of the drug, followed by disclosing any signs and symptom of their illness to the pharmacy professional (26.3%), providing a piece of paper with the name of the drug (11.3%) and taking a drug container to the drug retail outlet (7.5%) [Table 3].

When asked about the ailment that initiated the respondent to practice SM, the majority mentioned headache (25.7%). Abdominal pain and cough were the second and third most common causes of morbidity, with a frequency of 59 (12.9%) and 54 (11.89%), respectively. Other episodes of illness included diarrhea 43 (9.4%), toothache 39 (8.6), and stomach pain 29 (6.4%) [Table 3].

**Fig 2:**
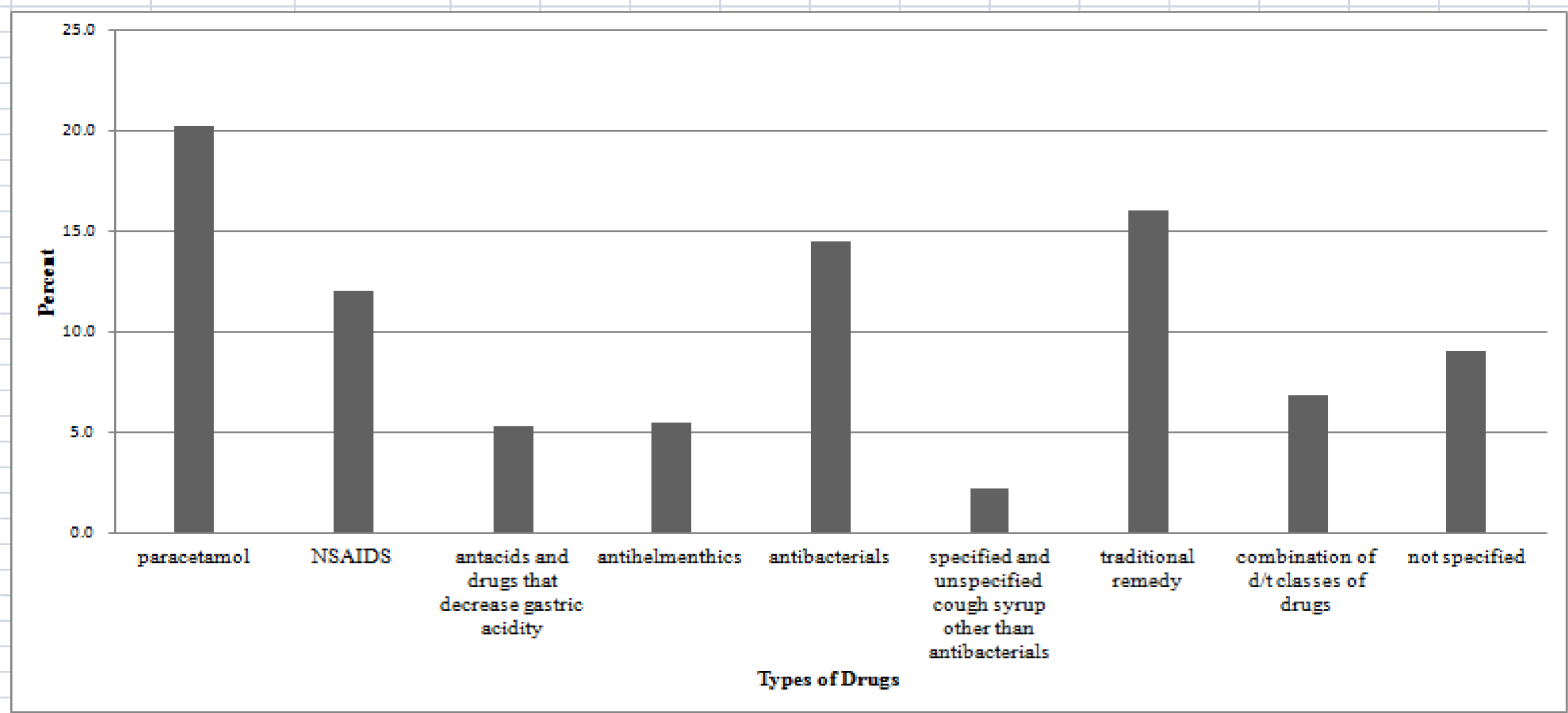
Frequency of drug/drug groups used among SM practiced study participants from April, 2016 to May, 2016.

Drugs or drug groups commonly used for SM among the 456 study participants who practiced SM are shown in Fig 2. Ninety two (20.2%) of those study participants who practiced SM were known to use paracetamol. Other medications used were traditional remedies constituting 73 (16.0%), followed by antibacterials 66 (14.5%), NSAIDs 55 (12.1%), anti-helminthes 25 (5.5%), and anti-acids and drugs that decrease gastric acidity 24 (5.3%).

Analyses done to determine the most frequently used individual drugs indicated the utilization of paracetamol by a 20.2% of those who practiced SM. Other drugs that were commonly used for the practice included traditional remedies, diclofenac, amoxicillin, metronidazole, and albendazole, constituting 16.0, 9.9, 6.6, 4.4 and 2.4%, respectively. A significant proportion (31.1%) of drugs used for SM was in the category of prescription drugs [Table 4].

**Table 4:** Most commonly used individual drugs for self-medication and prescription category of the drugs used in Addis Ababa from April, 2016 to May, 2016.

Drug/Drug type	Frequency	Percent
Paracetamol	91	20.0
Traditional remedies	73	16.0
Diclofenac	45	9.9
Amoxicillin	30	6.6
Metronidazole	20	4.4
Anti-acids	12	2.6
Albendazole	11	2.4
Mebendazole	9	2.0
Omeprazole	9	2.0
Ciprofloxacin	7	1.5
Niclosamide	6	1.3
Others*	143	31.4
**Prescription category of drugs used for SMP**		
OTC drug	162	35.5
Prescription drug	142	31.1
Traditional remedies	73	16.0
The drug used not specified	50	11.0
Combination of both prescription and OTC	29	6.4

*Indomethacin, ibuprofen, ampicillin, insulin, chloroquine, tinidazole, vitamin B, ketoconazole, acetylsalicylic acid, hyoscine, chloramphenicol eye drop, tetracycline eye drop, salbutamol, dextromethorphan, metformin, amlodipine, ranitidine, hydrochlorothiazide, prednisolone, almetamine, oral rehydration salt, combination of drugs.

Reasons for practicing SM were analyzed and shown in Table 5. Among the most common reasons for SMP were perception of mildness of illness (47.4%), previous knowledge about the medication (23.2%), and emergency use of the drug (10.5%).

**Table 5:** Reasons of study participants for practicing SM in Addis Ababa from April, 2016 to May, 2016.

Reason for SMP	Frequency	Percent
Minor illness	216	47.4
I know the drug before	106	23.2
Emergency case	48	10.5
Time constraint	28	6.1
Self-medication is cheap	25	5.5
I believe visiting health facility has nothing to add	10	2.2
Long wait at health facilities	10	2.2
Previous health institution visit has not produced any benefit	10	2.2
Health facility too far	3	0.7

**Fig 3:**
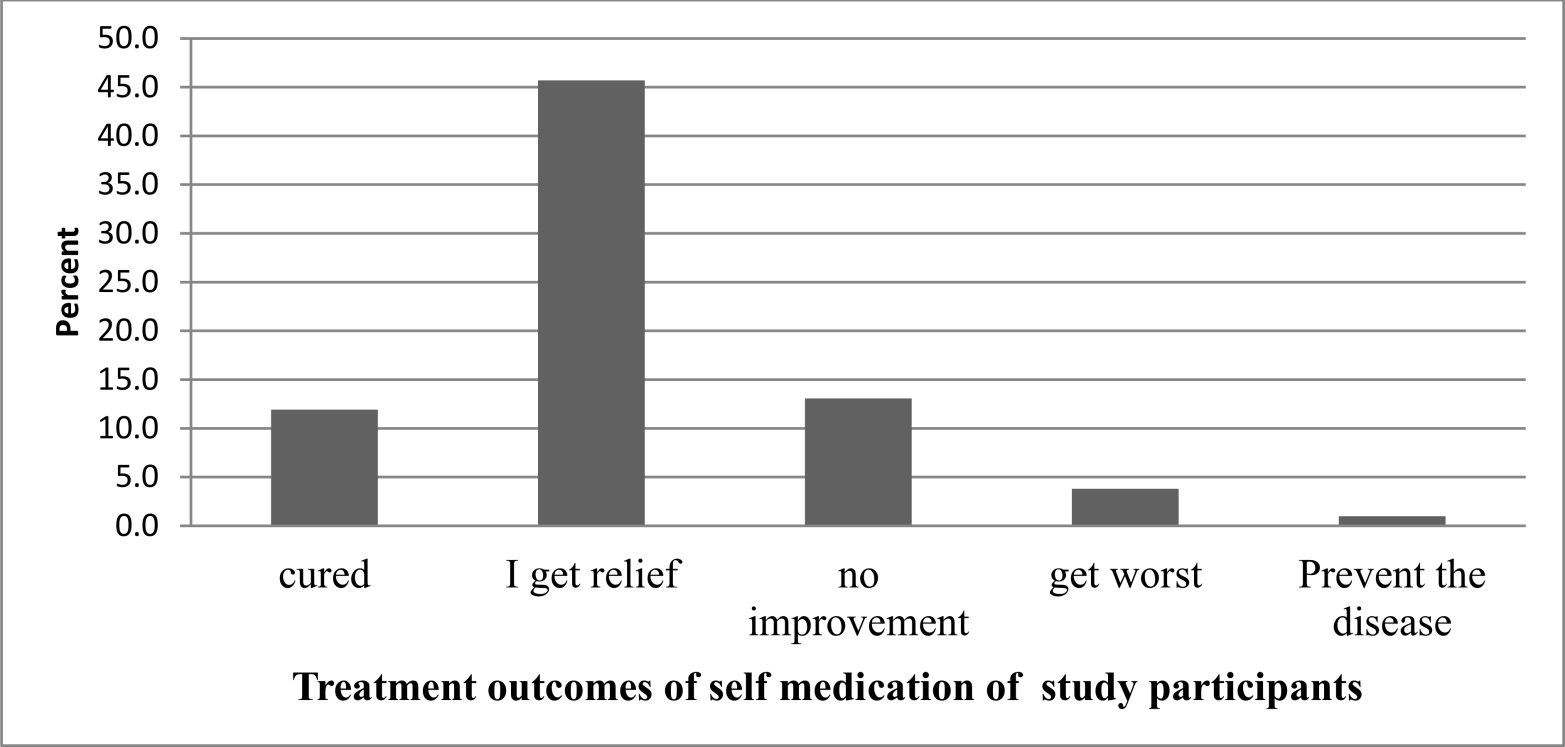
Treatment outcomes of SMP of study participants of Addis Ababa from April, 2016 to May, 2016.

This study also assessed the perceived outcome of the SMP. Two hundred and seventy five (45.5%) of those that practiced SM reported getting relief from the illness. Seventy nine (13.1%) reported no improvement. Seventy two (11.9%) said they got complete cure of their illness [Fig 3].

**Fig 4:**
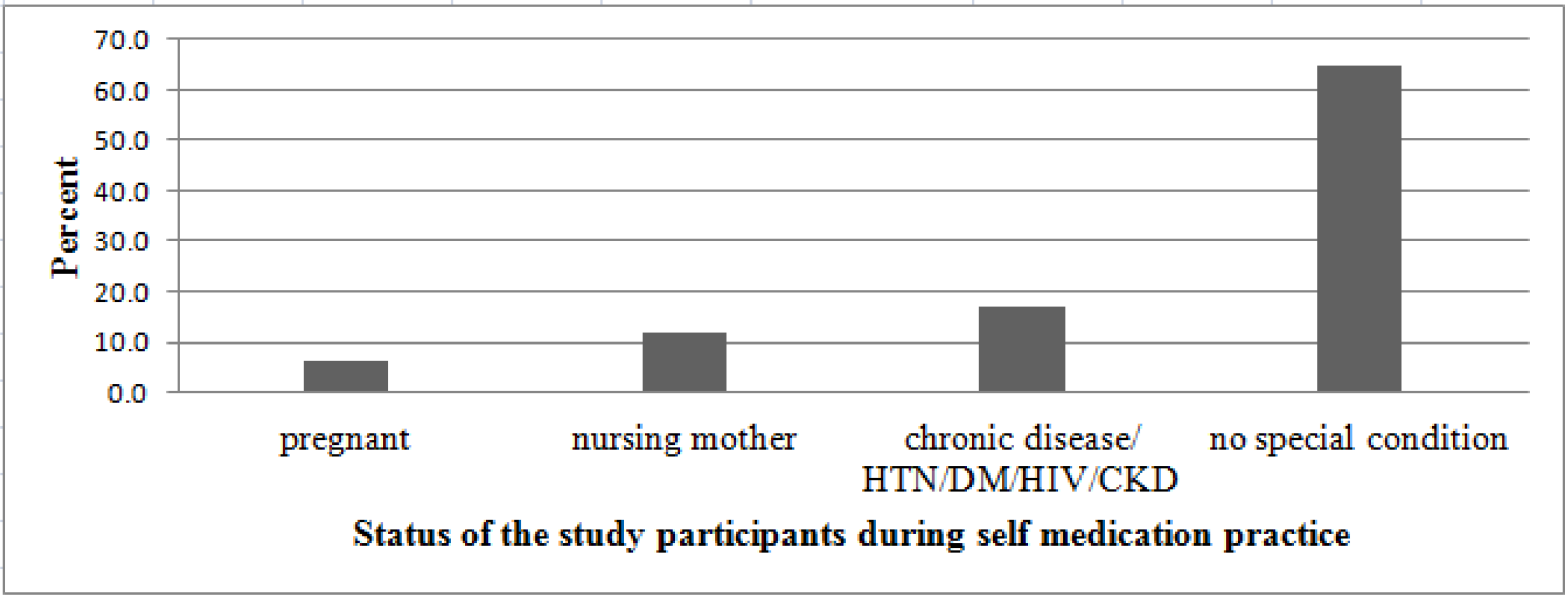
General status of SM practiced study participants in Addis Ababa from April, 2016 to May, 2016.

Those study participants who practiced SM were asked about presence of any special physiological and/or pathological condition during SM. The majority (64.7%) had no special condition. Fifty four (11.8%) were nursing mothers. Twenty nine (6.4%) were pregnant during SM. The rest (17.1%) had reported presence of chronic disease like hypertension, diabetes mellitus, HIV, and kidney disease [Fig 4].

### Knowledge about appropriate self-medication and attitude towards the practice

Thirteen questions were asked in order to assess the knowledge of the respondents about appropriate SM. Three hundred and seventy nine (62.7%) respondents said they knew some drugs should not be taken with other drugs simultaneously. Four hundred and eighty five (80.3%) said they knew the drugs that should not be taken with alcoholic drinks. Three hundred and seventy four (61.9%) reported they had adequate knowledge on the fact that some drugs should not be taken with certain food items. When asked whether the study participant knew which drugs should or should not be given to children, four hundred and forty eight (74.2%) said they knew which type of drugs those were [Table 6].

**Table 6:** Knowledge of study participants about appropriate SMP in Addis Ababa from April, 2016 to May, 2016.

Knowledge questions	Yes		No	
	N	%	N	%
Do you know availability of drugs that should not be simultaneously taken with other drugs?	379	62.7	225	37.3
Do you know availability of drugs that should not be taken with alcoholic drinks?	485	80.3	119	19.7
Do you know availability of drugs that should not be taken with certain types of food items?	374	61.9	230	38.1
Do you know availability of drugs that should not be given to children?	448	74.2	156	25.8
Do you know availability of drugs that should not be given to pregnant women?	441	73.0	163	27.0
Do you know availability of drugs that should not be taken by patients having chronic disease?	333	55.1	271	44.9
Do you know availability of drugs that should be taken by nursing mothers?	432	71.6	171	28.4
Do you know any drug that can be available in different dosage forms?	483	80	121	20
Have you the habit of discontinuing drug intake before the date advised by the health care provider?	132	31.8	412	68.2
Have you the habit of drinking alcohol while taking drugs?	115	19.0	489	81.0
Do you share drugs with family members, friends and neighbours?	153	25.3	451	74.7
Do you believe that a same drug can be a remedy and a poison?	367	63.7	237	39.2
Do you have the habit of checking expiry date of drugs during purchase or before use?	385	63.7	219	36.3

Four hundred and forty one (73.0%) and four hundred and thirty two (71.6%) study participants reported they knew which drugs should not be taken by pregnant and nursing mothers, respectively. Three hundred and thirty three (55.1%) study participants said they were aware of availability of drugs that should not be taken by patients having chronic diseases. Four hundred and eleven (68.2%) study participants reported they had no habit of discontinuing drug intake before the date advised by health care provider. The majority of study participants (81.0%) said they had no habit of taking drugs with alcoholic drinks. When asked whether or not the study participants had a habit of sharing drugs with family members, friends and/or neighbors, the majority (74.7%) said they had no habit of sharing [Table 6].

Study participants were asked about their belief regarding whether a same drug can be a remedy and a poison. The majority (60.81%) said they believe a drug can be a remedy and a poison. Three hundred and eighty five (63.7%) of study participants said they had habit of checking the expiry date of a drug before purchase and consumption [Table 6].

**Fig 5:**
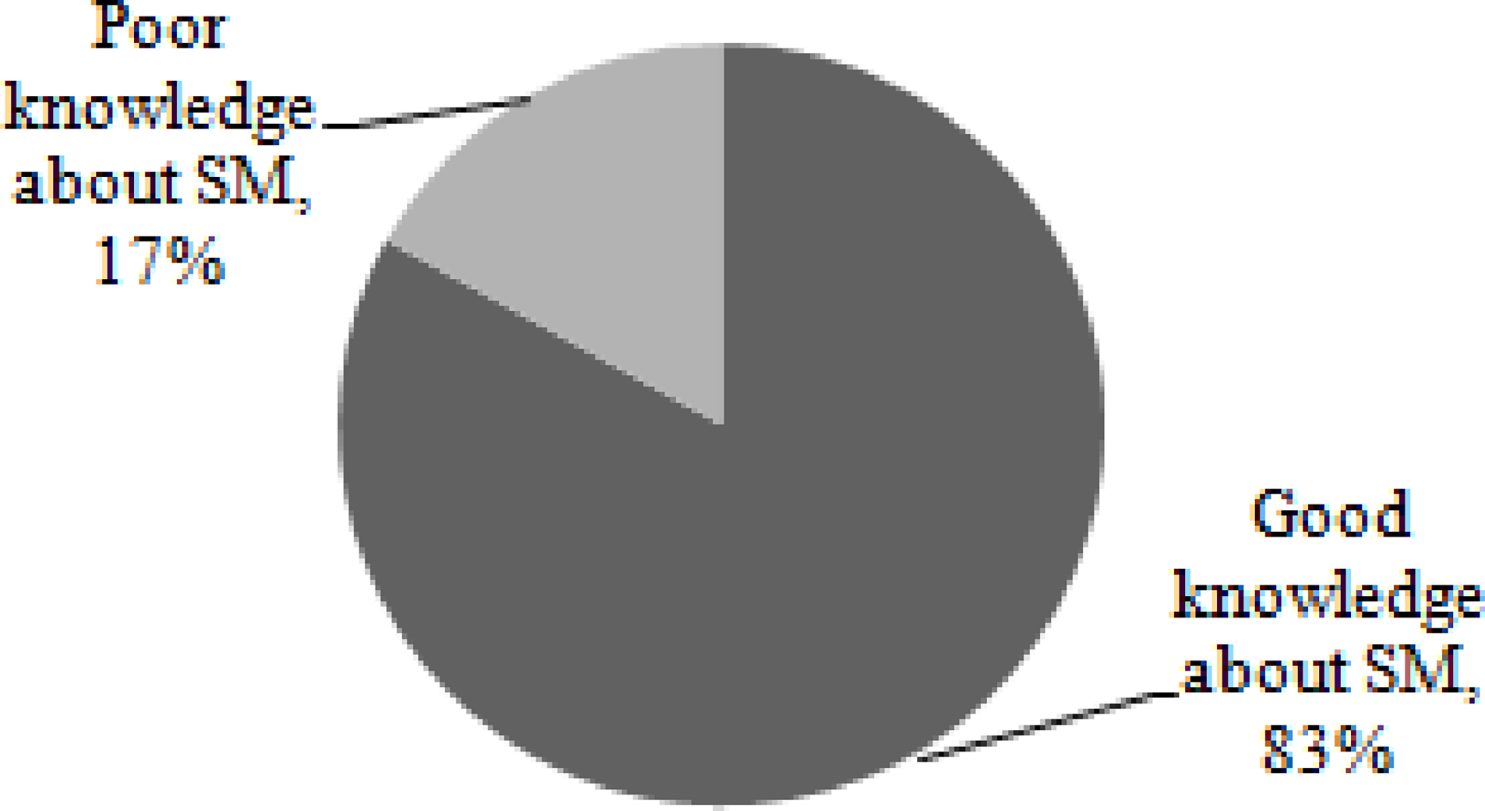
Knowledge of study participants about appropriate SMP in Addis Ababa from April, 2016 to May, 2016.

The knowledge of respondent about appropriate SMP was computed from the thirteen questions. The minimum and maximum scores to the questions were 1 and 13, respectively. The mean knowledge score was 9.07 with a standard deviation of 2.60. Finally, the overall knowledge category of study participants indicated that the majority of study participants (83.4%) had good knowledge about appropriate SM [Fig 5].

**Fig 6:**
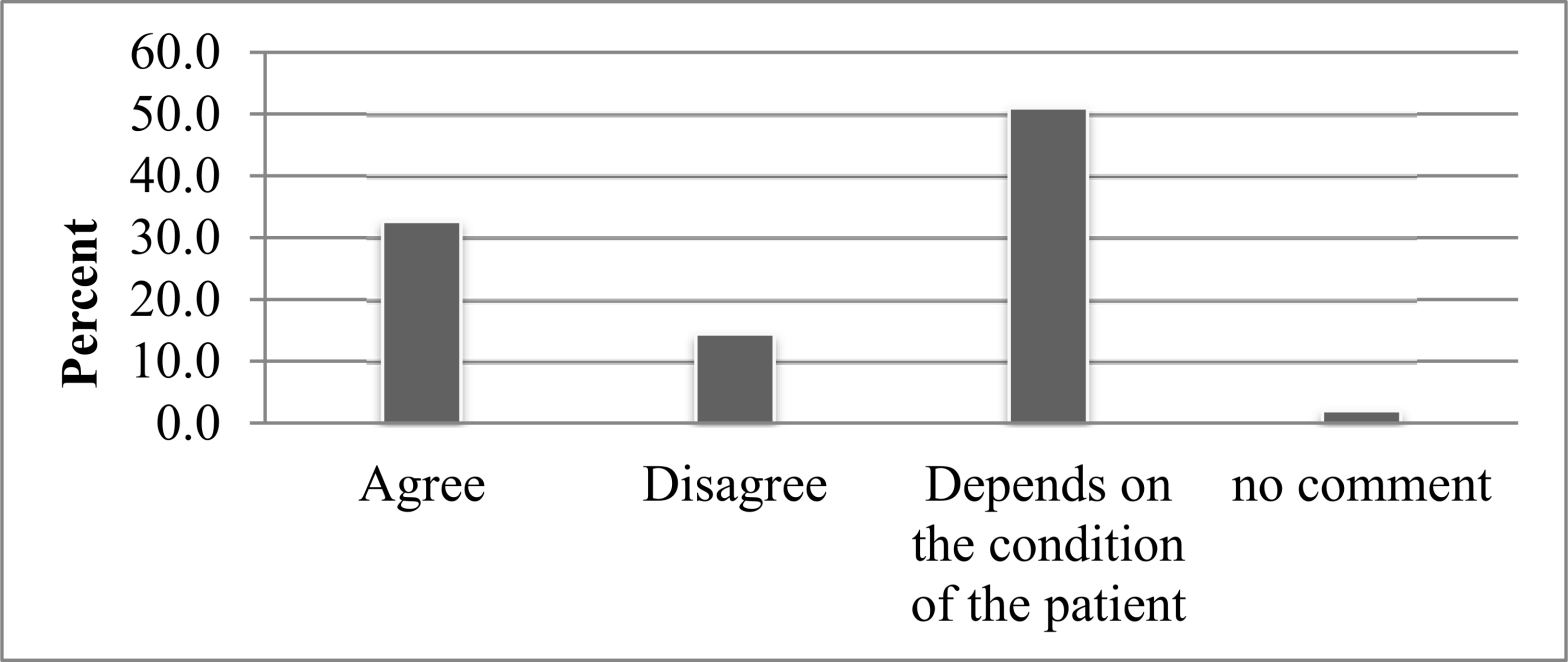
Attitude of study participants towards SMP in Addis Ababa from April, 2016 to May, 2016.

Study participants were asked to reveal their attitude towards SMP at the time of illness. The majority (51.0%) said they may agree or disagree depending on the condition and/or disease of the patient. One hundred and ninety seven (32.6%) agreed on the practice of SM without any condition. A very small proportion (2%) said they had no comment [Fig 6].

### Factors affecting self-medication practice

To identify factors affecting SMP, model chi-squares were utilized. According to the predicted probabilities, logistic regression was fitted.

On binary logistic regression analyses, the study participant’s income, age, and knowledge about appropriate SMP were associated with SMP. Those study participants at the age group of 25–34 were 0.52 times less likely to practice SM than those who are at the age group of ≥ 55 years (COR = 0.52[95% C.I:0.29–0.92]). Those study participants having a monthly income of 500–1000 birr were 1.67 times more likely to practice SM than those who had a monthly income of >1500 birr (COR = 1.67(95% C.I:1.05–2.65]). On the contrary, those study participants whose monthly income was 1001–1500 birr were 0.40 times less likely to practice SM than those whose monthly income was >1500 birr (COR = 0.40(95% C.I:0.19–0.84]) [Table 7].

**Table 7:** Factors affecting SMP in Addis Ababa, from April, 2016 to May, 2016.

Variables	SMP				Odds ratio	
	Yes		No		Crude	Adjusted
	N	%	N	%		
**Age**						
18–24	20	3.31	12	1.99	1.51(0.66–3.48)	1.55(0.65–3.71)
25–34	151	25.00	31	5.13	0.52(0.29–0.92)*	0.55(0.30–1.03)
35–44	125	20.70	32	5.30	0.64(0.36–1.15)	0.69 (0.37–1.26)
45–54	87	14.41	44	7.29	1.27(0.72–2.24)	1.34(0.74–2.41)
≥55	73	12.09	29	4.80	1	1
**Monthly income (birr)**						
<500	98	16.22	37	6.13	1.27(0.79–2.06)	1.00(0.59–1.69)
500–1000	93	15.39	76	12.58	1.67(1.05–2.65)*	1.4(0.85–2.29)
1001–1500	76	12.58	9	1.49	0.40(0.19–0.84)*	0.34(0.16–0.74)*
>1500	189	31.29	56	9.27	1	1
**Sex**						
Female	325	53.81	97	16.06	1.30(0.88–1.94)	0.75(0.49–1.17)
Male	131	21.69	51	8.44	1	1
**Knowledge status of respondent**						
Good knowledge	392	64.90	112	18.54	1	1
Poor knowledge	64	10.59	36	5.96	1.97(1.24–3.12)*	2.04(1.24–3.33)*

*There is statistically significant association with p≤0.05.

This study also indicated that those study participants who had poor knowledge about appropriate SM were 1.97 times more likely to practice SM than those who had good knowledge (COR = 1.97[95% C.I:1.24–3.12]) [Table 7].

Finally, after obtaining statistically significant variables at p≤ 0.05 in binary logistic regression analyses, multivariable logistic regressions were carried out to see the independent predictors of SMP of respondents. After adjusting for potential confounders, the monthly income of respondent and knowledge about appropriate SMP were found to be independent predictors of SMP. Accordingly, those study participants whose monthly income was 1001–1500 birr were less likely to practice SM than those whose income was >1500 birr (AOR = 0.34[95% C.I:0.16–0.74]). Those study participants who had poor knowledge about appropriateness of SM were 2.04 times more likely to practice SM than those who had good knowledge (AOR = 2.04[95% C.I:1.24–3.03]) [Table 7].

## Discussion

This study attempted to assess self-medication practice among Addis Ababa community residents and further analyzed different factors that affect the practice such as, illness for which drug was used, the pharmacologic category of drug utilized, the reason to practice SM, and perceived outcome of the SM.

The prevalence of SM in this study was found to be 75.5%. Headache 117 (25.70%), abdominal pain 59 (12.90%), and cough 54 (11.8%) were the three most frequently reported ailments. The two main reasons for SM were mildness of illness 216 (47.40%) and previous knowledge about the drug 106 (23.20%). Paracetamol 92 (20.20%) and traditional remedies 73 (16.0%) were the two most frequently consumed medications.

According to the findings of this study, 75.5% of the study participants had practiced SM. This is by far greater than those findings reported from Sire town (27.26%), Nekemte town (36.7%), Jimma town (27.6%), Assendabo town (39%), and Kolladiba town (62.8%) [4, 9, 13, 14, 15]. SMP is also more prevalent in this study in comparison to reports from students of Mekelle University (42.67%) and students of University of Gonder (38.5%) [10, 16]. This variation might be due to the difference in the study population, the fact that those study participants who used traditional remedies including herbs for SM were included as practitioners of SM in the current study and availability of a plethora of information and drug retail outlets in the capital city of Addis Ababa. Various studies conducted in different locations of the world has shown a range of SM practices between 8.3 to 87% [7,8,17, 18, 19, 20, 21, 22, 23, 24]. The reasons for this wide variation might be differences in socio-demographic characteristics of the study participants including academic status, non-availability of medical facilities in some areas, easy availability of drugs, and types of drugs that the study intends to identify.

The findings of this study indicated that there is a statistically significant association between monthly income and SMP. Those study participants whose monthly income was 1001–1500 birr were less likely to practice SM than those whose monthly income was >1500 birr (AOR = 0.34 (95% C.I:0.16–0.74). This is contrary to a study from Kolladiba in which female respondents (70.1%), married respondents (65.2%), respondents with the level of secondary education (35.4%), respondents who were merchants (34.7%) and respondents who had very low income (49.4%) were found to practice self-medication more than their counterparts from their respective categories [15]. The finding of the current study is also not in line with a study finding from Sudan where the middle-income group was found to be more self-medicating and from a study in Sire town where no association was obtained between SMP with sex, educational status, occupation, and income [14, 25]. The reason might be that the low-income group in this current study could not afford the cost of medication, that the city administration would provide free medical service for those who could not afford the cost of health care, and because of differences in categorization of income categories.

The current study indicated that three hundred and five (50.5%) study participants had SMP with modern medicines. The rest used either traditional medicine alone or in combination with modern medicine for SM purpose. This finding is almost comparable to the finding from Kolladiba town where 51.8% of study participants used modern drugs for SM [15]. However, in a study conducted in Western Nepal, only 8.7% of participants practiced SM with herbal remedies [26]. The very large magnitude of involvement of traditional medicine for SM in our study and the Kolladiba study indicates a great deal of attention needed to be given to this area of medicine in the country.

The kind of sources of information on modern drug used for SM in this study was similar to that mentioned in studies conducted among communities in Mekelle town, health science students from University of Gonder, students from Mekelle University, on the urban slum community of India, residents from Assendabo town, and in many others studies (although it differs in magnitude of the source) [4, 7, 10,16, 27].

Drug retail outlets were identified as the main source of modern medicine used for SM in this study. This is also the case in Sire, Mekelle and Kolladiba towns [14, 15, 27]. Moreover, significant proportions (31.1%) of modern drugs used for SM in this study were prescription only drugs among the total self-medicated drugs. The reasons for this might be a weak enforcement of regulations regarding drug handling and dispensing [4]. This study also indicated the possibility of obtaining drugs from neighbors and through remnants from previous treatment which implies the use of low quality drugs, development of resistance to antimicrobials, and treatment failures. Delivering health education on the appropriate use of drugs to the community may alleviate these problems.

We found that the most common types of ailments that led to SM in this study were headache followed by abdominal pain, cough, diarrhea, and toothache. In a study conducted a few years ago among residents of Addis Ababa, the three most commonly reported illnesses for which study participants sought SM were gastrointestinal disease, headache/fever and respiratory problems [28]. A similar study conducted in Sire town reported headache, cough and cold, and diarrhea as the three most commonly occurring illnesses that led to SM [14]. Headache and fever, respiratory tract infections and gastrointestinal disorders were also the three most common ailments that became reasons for SM in the Kolladiba study [15]. More or less similar ailments with different magnitude were mentioned as reasons for practicing SM in students from Mekelle University, residents of Assendabo town, University of Gonder students, residents of Jimma town, urban slum areas of India, university students of Brazil, and in many communities [4, 7, 9, 10, 16, 19, 26]. Thus, as reported by the different findings, headache and cough were the most common ailments for which study participants would get treatment via SM.

The most commonly employed drug/drug classes for SM in this study were paracetamol, followed by traditional remedies, NSAIDs, antibacterials, anthelmintics and antiacids and other drugs that decrease gastric acidity. That analgesics are the most commonly employed drugs for SM in this study is in line with study findings from Addis Ababa (where analgesics/antipyretics, antimicrobials and gastrointestinal drugs were the three most commonly employed drug categories for SM), Kolladiba town (where analgesics, antibiotics and GI drugs were reported as the three most commonly employed drugs categories for SM), Sire town (where the three most commonly employed drug categories for SM were analgesics, antibiotics and traditional remedies) and Mekelle town (where analgesics, gastrointestinal drugs and respiratory drugs were the three most commonly employed drug categories for SM) [14,15,27,28]. Similar drug/drug classes with different magnitude were mentioned in studies conducted in Nepal, and among students of University of Gonder and Mekelle Universities. In the Nepal study, paracetamol, NSAIDs, cold remedies, antiacids, and herbs were the most commonly employed drug/drug classes for SMP [26]. A study conducted among students of University of Gonder pointed out paracetamol, NSAIDs, antacids, anthelmenthics, antibiotics and antimalarial drugs as the most commonly used drugs for SM [10]. The most commonly used drugs for SM reported in the study conducted on students from Mekelle University were paracetamol, NSAIDs, antibiotics, cough syrup, and antiacids [16].

This study indicated that the most commonly employed individual drugs were paracetamol, diclofenac, amoxicillin, Metronidazole, and albendazole. According to this study, a significant proportion of study participants (20.0%) employed antimicrobials for SM. Use of antimicrobials for SM was also seen in different studies conducted in different parts of the country. For example, 24.10%, 24.70% and 33.00% of study participants used antimicrobials for SM in Sire, Kolladiba, and Mekelle studies, respectively [14, 15, 27]. One major problem with SM with antimicrobials is the emergence of drug resistant pathogenic microbes [25]. The use of antimicrobial agents for SM was also reported and mentioned as one public health problem in other studies conducted in Mekelle University, Gonder University, Jimma town, Iran, Bahrain, and Sudan, among others [9,10,16, 21, 24, 25, 29]. Thus, use of prescription drugs without prescription should be discouraged and appropriate health education should be provided by all concerned bodies in order to raise the awareness of the society on appropriate utilization of drugs in general and antimicrobials in particular.

The way of requesting drugs by the participants who obtained drugs from drug retail outlets in this study was by mentioning the name of the drug in majority of the cases followed by telling symptoms of illness to the pharmacy professional and by showing a piece of paper with the name of drug. The majority of self-medicating individuals (57.4%) in the Addis Ababa study indicated a similar way of drug request for SM as the current study [28]. Similar ways of requesting drugs were obtained in a study conducted at Mekelle town where the majority (20.8%) requested drugs by telling illness symptoms [27]. A study conducted in urban slum areas of India indicated the majority of respondents requested by explaining symptoms of illness [7].

Unlike the findings from the study in Assendabo town, where the majority of participants who practiced SM reported worsening of their condition, in our study the majority (60.5%) reported relief from their illness. In contrast to the finding of our study, 82.3% of study participants who practiced SM in the Mekelle study showed improvement in their disease symptoms due to use of the drug [27].

Regarding reason of respondents for choosing SM, the majority of study participants in the current study pointed perception of mildness of illness (47.4%) followed by previous knowledge about the medication (23.2%) and emergency use of the drug (10.5%). This finding is more or less similar to the finding from Sire town where study participants mentioned reduced cost of SM, mildness of illness, the emergency nature of the case, and previous experience with drug used as the top four reasons for SM [14].

The current study indicated that those participants who had poor knowledge about appropriate SM were 2.04 times more likely to practice SM than those who had good knowledge (AOR = 2.04[95% C.I:1.24–3.03]). The reason for this might lie in the fact that those individuals who had good knowledge about appropriate SMP prefer to consult a physician before practicing SM.

The findings of this study indicated that a significant proportion of self-medicating individuals were pregnant (6.4%), nursing mothers (11.8%) or had a chronic disease(s) (17.1%). This is in line with the finding from Addis Ababa where a significant percent of SM individuals were Pregnant (6.1%), breast-feeding (6.7%), or had a chronic disease(s) (87.2%) [28]. This might be due to reduced awareness or lack of knowledge of participants about teratogenic potential of drugs, effect of drugs on nursing an infant and the effect of drugs on other drugs that are taken for treatment of other diseases.

In this study, the majority of participants (51.0%) said they may agree or disagree to practice SM depending on the illness and the drug used for treatment followed by agree (32.6%) and disagree (14.4%) with SMP. Even though this finding is contrary to the finding from Gonder University students where 55.5% agreed with SMP, it is in agreement with the finding from Mekelle University students where only 36.70% agreed to the practice of SM [10, 16]. The finding from a study in Bahrain indicated that the majority (76.9%) of respondents had a positive attitude favoring SM [21]. This difference in attitude towards SM from place-to-place might be due to differences in access to information about medicines, utilization of different types of study participants and access to medicines.

## Strength of the study

The strengths of this study were having an adequate sample size that could represent the Addis Ababa community by using appropriate sampling techniques, and utilization of appropriate statistical methods and tests to minimize biases and analyze the data.

## Limitations of the study

Recall bias and provision of socially acceptable responses by the study participants, and potential variation in interviewer techniques that lead to unique responses were the possible limitations of this study. To minimize the recall bias, proper definition and articulation of the research question, administering the interview properly and consistently, and shortening the recall period to two months were done. To minimize the possibility of providing socially acceptable responses, the study participants were assured about the confidentiality of the information they provide, and declared in the research consent form every answer they provide was valuable. Moreover, potential variation in the interviewer technique that might have elicited unique responses was minimized by assigning supervisors to follow the data collection process, and make corrections for any errors. Questions were also made possibly neutral in tone and placed in logical order, and interviewers’ were advised to continually practice and refine their interviewing techniques.

## Conclusion

The majority of the study participants had practiced self-medication. Their common reasons for this practice were mildness of illness, previous knowledge about the drug, and emergencies of the illness. The most typical sources of drug information about the available modern drugs for SMP were consultation by a health professional, drug experience from previous treatment, and friends. Drug retail outlets were the main sources for obtaining drugs.

Headache, abdominal pain, cough, diarrhea and toothache were the main ailments of the participants that took to SM and their respective frequently used drugs were paracetamol, traditional remedies, NSAIDs, antibacterials, and antihelmintics. Monthly income and knowledge about appropriate SM were both significantly associated with SMP.

## Recommendations

Although appropriate SMP is one of the components of self-care adopted by the WHO, its irrational use is very likely to bring serious health consequences as observed in this current study. Therefore, based on the findings of this study, we recommend:

- That drug regulatory and health authorities allocate some resources in order to:
  ○ Provide health information on SMP: proper time, type of drugs, and advantages and disadvantages of SM.
  ○ Enforce rules determining drug prescription and dispensing.
  ○ Increase awareness among community members on antimicrobial drug resistance and its public health impact.
- To conduct further studies to be performed in various seasons and for longer periods.
- Active participation of pharmacy professionals to fight against the negative consequences of SMP and educate people looking for SM.

## Supporting information

S1 File Data file. (SAV)

S2 File Questionnaire for self-medication practice (English). (DOCX)

S3 File Questionnaire for self-medication practice (Amharic). (DOCX)
