# Supplementary material for: Prevalence and determinants of self-medication practice among selected households in Addis Ababa community
Source: PLoS One. 2018 Mar 26;13(3):e0194122. doi: 10.1371/journal.pone.0194122 (PMC5868796; doi:10.1371/journal.pone.0194122)
Supplement: S2 File — (DOCX) [file pone.0194122.s002.docx]

## Questionnaire

The aim of this study is to measure the prevalence of self-medication practice; assess the knowledge about appropriate self-medication practice; identify common diseases and drugs; and factors affecting self-medication in Addis Ababa. The information that you provide has great contribution and we would like to promise that all information will be kept confidential and for the current research purpose only. Therefore we kindly request you to answer what you righteously feel without any fear while the interviewer asks.

Would this be okay with you?

I understand about the advantage of the research and the roles I will have in the research. I have agreed to participate in the research.

Yes B. No

1. **Socio-Demographic Data**
2. Age: __________
3. Gender:
4. Male
5. Female
6. Religion
7. Orthodox Christian
8. Muslim
9. Protestant
10. Others (please specify): __________
11. Marital status
12. Single
13. Married
14. Divorced
15. Separated
16. Ethnicity
17. Amhara
18. Oromo
19. Tigray
20. Others (please specify): ________
21. Educational Status
22. Illiterate
23. Read and write but have no formal education
24. Elementary
25. Secondary school
26. Higher education
27. Income level per month (in Birr):________________
28. Occupation

A. Student

B. Government employed

C. Employed in private business

D. Have private owned business

E. House wife

F. Others ___________________

1. Family Status
2. Father
3. Mother
4. Child
5. Relative
6. **Questions related to self-medication**
7. Have you ever self-medicated within the past two months?

A.yes B.No

1. If No to Q1 what was your reason?
   1. Not to use wrong drug
   2. Fear of side effects
   3. Fear of wrong diagnosis
   4. Fear of wrong use of drug
   5. I had no illness in the specified time
   6. Others
2. If “yes” to Q1, what type of medication do you use?
3. Modern medicine
4. Traditional medicine
5. Both type
6. If your answer for Q3 is modern medicine, what was your source of information about the drugs?
7. Health professional
8. Experience from previous treatment
9. Friend
10. Book/internet
11. Others, please specify: ___________________
12. If your answer is “modern medicine” for Q3, where do you get the drugs for self-medication?
13. From pharmacies/drug shops
14. Left over from previous treatment
15. From neighbor
16. Others, please specify: __________________
17. How do you request the drug if the source for the drug is drug retail outlets?
18. By mentioning the name of drug
19. By mentioning the sign and symptom of illness
20. By Showing drug container
21. By showing a piece of paper on which the name of the drug is written
22. Others; please specify________________________
23. What was the ailment that lead you practice self-medication?
24. Headache
25. Fever
26. Cough
27. Abdominal pain
28. Toothache
29. Diarrhea
30. Peptic ulcer disease
31. Eye disease
32. Constipation
33. Other (specify)________
34. Could you tell/show me the drug/drugs that you used for self-medication purpose?

_____________________________________

1. Can you please tell me why you practice/prefer self-medication?
2. Time constraint
3. Minor illness
4. Health institution is too far
5. Emergency case
6. Self-medication is cheap
7. I know the drug before
8. Others (please specify): _____________________________________
9. What was the perceived outcome of the self-medication?
10. Cured from the illness
11. Get relief from the illness
12. No improvement
13. Get worsened
14. Others (Please specify):______________________________
15. Was there any special physiologic/pathologic condition while self-medicating?
16. No special condition
17. Pregnant
18. Breastfeeding
19. Had a chronic disease such as diabetes, hypertension, liver disease, kidney disease, etc
20. Other? Specify
21. **Questions related to knowledge of participant about appropriate SMP?**
22. Do you know that some drugs:
23. Cannot be taken with other drugs? A. Yes, I know B. No, I don’t know
24. Cannot be taken with alcoholic drinks? A. Yes, I know B. No, I don’t know
25. Cannot be taken with some kinds of foods? A. Yes, I know B. No, I don’t know
26. Do you know that some drugs are contraindicated or
27. Cannot be given to children? A. Yes, I know B. No, I don’t know
28. Cannot be given to pregnants? A. Yes, I know B. No, I don’t know
29. Cannot be given to breast feeding mothers? A. Yes, I know B. No, I don’t know
30. Cannot be taken by people with chronic diseases? A. Yes, I know B. No, I don’t know
31. Do you know that the same drug can be given by oral, injection, topical or other routes?
32. Yes, I know B. No, I don’t know
33. Did you discontinue taking drugs before the date advised by the health care provider?
    1. Yes B. No
34. While taking drugs, do you usually take alcoholic drinks?
35. Yes B. No
36. Do you share drugs with family members, friends, neighbors, etc?
37. Yes B. No
38. Do you believe that the same drug can be a remedy or a poison?
39. Yes B. No
40. Do you check expiry date of drugs during purchasing or before use?
41. Yes B. No
42. Would you tell us your attitude towards self-medication practice?
    1. Agree
    2. Disagree

C. Depends on the type of disease to be treated and the drug to be used

D. No comment
