# Supplementary material for: Prevalence and determinants of self-medication practice among selected households in Addis Ababa community
Source: PLoS One. 2018 Mar 26;13(3):e0194122. doi: 10.1371/journal.pone.0194122 (PMC5868796; doi:10.1371/journal.pone.0194122)
Supplement: S3 File — (DOCX) [file pone.0194122.s003.docx]

# የመጠይቁ መለያ ቁጥር፡ __________

**የጥናት ቃለ-መጠይቅ ቅጽ**

**የጥናቱ ርእስ፡** **በአዲስ አበባ ከተማ ነዋሪዎች ውስጥ ራስን በራስ በመድሀኒት** የ**ማከም ልማድና ተያያዥ ጉዳዮችን ማጥናት**

የዚህ ጥናት ዋና አላማ ***በአዲስ አበባ ከተማ ነዋሪዎች ውስጥ ያለውን ራስን በራስ በመድሀኒት የማከም ልማድና ተያያዥ ጉዳዮችን ማጥናትና*** መለየት ነው፡፡ የሚሰጡን መረጃ ጥናቱን የተሟላ ከማድረጉም በተጨማሪ የህብረተሰቡን የጤና ችግር ከመቅረፍ አንጻር ትልቅ አስተዋጽዖ ይኖረዋል፡፡ በዚህ ጥናት ላይ ለመሳተፍ ከተስማሙ ይህ ግለሰባዊ መረጃ ለጥናቱ ብቻ የሚውል ነው፡፡ ግልጽ ያልሆነ እና ማብራሪያ የሚያስፈልግዎ ከሆነ ምንም ሳያመነቱ አስተባባሪውን መጠየቅ ይችላሉ፡፡

አሁን በጥናቱ ለመሳተፍ ፍቃደኛ ነዎት? ሀ. አዎ ለ. አይደለሁም

በጥናቱ ለመሳተፍ ፍቃደኛ ከሆኑ በፊርማዎ ያረጋግጡልን፡፡ ፊርማ ________________

ለፍቃደኝነትዎ እናመሰግናለን!

**ክፍል አንድ ፡ ማህበራዊ ጉዳዮችን የተመለከቱ ጥያቄዎች**

1. እድሜዎ ስንት ነው? ____________________
2. ጾታ ሀ. ሴት ለ. ወንድ
3. ሃይማንዎትዎ ምንድን ነው? ሀ. ኦርቶዶክስ ለ. ሙስሊም ሐ. ፕሮቴስታንት መ. ሌላ (ይግለጹት __________)
4. የትዳር ሁኔታ ሀ. ያላገባ ለ. ያገባ ሐ. የፈታ /በሕግ መ. የተለያዩ /የተራራቁ
5. ብሔርዎ ምንድን ነው? ሀ. አማራ ለ. ኦሮሞ ሐ. ትግራይ መ. ሌላ (ይግለጹት_____________)
6. የትምህርት ደረጃ

ሀ. ያልተማረ

ለ. መጻፍና ማንበብ

ሐ. የመጀመርያ ደረጃ

መ. ሁለተኛ ደረጃ

ሠ. ከፍተኛ ደረጃ

1. ስራ

ሀ. ተማሪ

ለ. የመንግስት ሰራተኛ

ሐ. በግል የስራ ዘርፍ የተቀጠረ

መ. በራሱ የግል ስራ የተሰማራ

ሠ. ቤት ስራተኛ

ረ. ሌላ (ይግለጹት_____________)

1. የቤተ-ሰብ ሁኖታ

ሀ. አባት

ለ. እናት

ሐ. ልጅ

መ. ቤተ-ሰብ

1. ወርሐዊ ገቢ (በብር)፡__________

**ክፍል ሁለት፡ “ ራስን በራስ ማከምን በተመለከተ የሚሰበሰብ መረጃ”**

1. ባለፉት ሁለት ወራት ውስጥ ታመው ራስዎን በራስዎ አክመዉ ያውቃሉ? ሀ. አዎ ለ. አላዉቅም
2. ለጥያቄ ቁ-1 መልስዎ አላዉቅም ከሆነ፤ ምክንያትዎን ቢገልፁልን?

ሀ. የተሳሳተ መድሃኒትን እንዳልጠቀም

ለ. የመድሃኒት የጎንዮሽ ጉዳት በመፍራት

ሐ. በሽታውን በትክክል መለየት ስለሚከብድ

መ. መድሃኒቱን በተሳሳተ ሁኔታ እንዳልጠቀም

ሰ. ሌላ ካለ ቢገልፁልን ________________

1. ለጥያቄ ቁ-1 መልስዎ አዎ ከሆነ፤ የምን ዓይነት ህክምና መድሐኒት ነበር የተጠቀሙት?

ሀ. የዘመናዊ ህክምና መድሐኒት ለ. የባህላዊ ህክምና መድሐኒት ሐ. የሁለቱንም ዓይነት ህክምና መድሃኒት

1. ለጥያቄ ቁ-3 መልስዎ የዘመናዊ ህክምና መድሐኒት ከሆነ፤ ስለተጠቀሙት መድሐኒት **መረጃውን** ከየት ነበር ያገኙት?

ሀ. ከጤና ባለሙያ ለ. ከበፊት ህክምና ሐ. ከጓደኛ መ. ከመፅሐፍ/ኢንተርኔት ረ. ከሌላ (ግለፅ)_______________________

1. ለጥያቄ ቁ-3 መልስዎ የዘመናዊ ህክምና መድሐኒት ከሆነ፤ መድሐኒቱን **ከየት** ነበር ያገኙት?

ሀ. ከፋርማሲ/ ከመድሐኒት መደብር

ለ. ከሌላ ሕክምና ከተረፈ መድሐኒት

ሐ. ከጎረቤት መ. ከሌላ (ግለፅ)_______________________

1. መድሐኒቱን የገዙት ከፋርማሲ ከሆነ እንዴት ነበር የጠየቁት?

ሀ. የመድሐኒቱን ስም በመጥቀስ

ለ. የሕመሙን ምልክት በመጥቀስ

ሐ. የበፊት የመድሐኒቱን መያዥያ በመያዝ

መ. በብጣሽ ወረቀት የመድሐኒቱን ስም በመያዝ

ሠ . ሌላ (ይገለፅ) _______________________

1. መድሐኒቱ የተጠቀሙት **ምን ለማከም ነበር?**

ሀ. ራስ ምታት

ለ. ትኩሳት

ሐ. ሳል

መ. ለሆድ ህመም

ሠ. ጥርስ ህመም

ረ. ተቅማጥ

ሰ. የጨጓራ ሕመም

ቀ. ለዓይን ህመም

ሸ. ለሆድ ድርቀት

ኘ. ሌሎች /ግለፅ/ __________

1. ራስዎን በራስዎ ለማከም የተጠቀሙትን ዘመናዊ መድሃኒት ቢጠቅሱልን ?

______________________________________________________________________________________________________________________________

1. ራስዎን በራስዎ እንዲያክሙ ያነሳሳዎ ምክንያት ምንድን ነበር?

ሀ. መታከሚያ ጊዜ ስለሌለኝ

ለ. ቀላል በሽታ ስለሆነ

ሐ. የጤና ተቋሙ ሩቅ ስለሆነ መ. ህመሙ ድንገተኛ ስለሆነ

ሠ. ርካሽ ስለሆነ

ረ. መድሐኒቱን ስለማዉቀዉ

ሰ. ሌላ ምክንያት ካለ (ቢገልፁልን)_________

1. ራስዎን በራስዎ ሲያክሙ ውጤቱ ምን ነበር?

ሀ. ከህመሙ መሉ ለሙሉ ድኛለሁ

ለ. ሕመሙ ተሽሎኛል

ሐ. አልተሻለኝም

መ. ሕመሙ ባሰብኝ

ረ. ሌላ (ግለፅ ) _______________________

1. ከህመሙ በተጨማሪ መድሐኒቱን ሲጠቀሙ የነበሩበት ሁኔታ ከነበረ ቢገልፁልን? ለምሳሌ፡ ሀ. የተለየ ነገር አልነበረኝም ለ. እርጉዝ ነበርኩ ሐ. አጠባ ነበር መ. ለመዳን የሚያስቸግሩ በሽታዎች (የደም ግፊት መጨመር፣ የሥኳር በሽታ፣ የኩላሊት በሽታ፣ወዘተ) ታማሚ ነበርኩ ሠ. ሌላ (ቢገልፁልን)_______________________

**ክፍል ሶስት፡ ተጠቃሚዉ ስለመድሀኒት ያለዉን እዉቀት መለኪያ ክፍል**

1. አንዳንድ መድሀኒቶች ከሚከተሉት ነገሮች ጋር በተመሳሳይ ሰአት እንደማይወሠዱ ያዉቃሉ?

ሀ. ከሌሎች መድሀኒቶች ጋር አዉቃለሁ_______ አላዉቅም______________

ለ. ከአልኮል መጠጦች ጋር አዉቃለሁ____________ አላዉቅም______________

ሐ. ከአንዳንድ አይነት ምግቦች ጋር አዉቃለሁ______ አላዉቅም___________

1. አንዳንድ መድሀኒቶችን የሚቀጥሉት አይነት ሠዎች እንደማይወሥዷቸዉ ያዉቃሉ?

ሀ. ህፃናት አዉቃለሁ_______________ አላዉቅም______________

ለ. ነፍሰጡርና አዉቃለሁ____________ አላዉቅም________________

ሐ. የሚያጠቡ እናቶች አዉቃለሁ____________ አላዉቅም__________

መ. አንዳንድ ለመዳን የሚያስቸግሩ በሽታዎች (የደም ግፊት መጨመር፣ የሥኳር በሽታ፣ የኩላሊት በሽታ፣ወዘተ) ታማሚ  ሠዎች አዉቃለሁ______ አላዉቅም________

1. አንድ መድሀኒት በተለያየ መንገድ ለምሳሌ በቅባት መልክ ፤በመርፌ ፤በኪኒን በሽሮፕ ወዘተ ሊሠጥ እንደሚችል ያዉቃሉ? አዉቃለሁ________________ አላዉቅም_____________
2. መድሀኒቶችን መዉሰድ ከጀመሩ በኋላ የጤና ባለሙያዉ ዉሰድ ካለዉ ጊዜ ቀድመዉ አቋርጠዉ ያዉቃሉ? አዉቃለሁ________________ አላዉቅም_____________
3. መድሀኒት በሚወስዱበት ጊዜ የአልኮል መጠጥ ይጠጣሉ?

ሀ. አዎ ለ. አልጠጣም

1. የሚወስዱትን መድሀኒት ለቤተሰብ አባል ፤ ለጓደኛ ፤ለጎረቤት ይሠጣሉ (ያጋራሉ)?

ሀ. አዎ ለ. አልሠጥም

1. አንድ መድሀኒት በሽታን የሚያድነዉን ያክል ከባድ ጉዳት ሊያመጣ (መርዝ ሊሆን) እንደሚችል መረጃ አለዎት?

ሀ. አዎ ለ. የለኝም

1. መድሀኒቶችን ከመግዛትዎም ሆነ ከመጠቀምዎ በፊት የመጠቀሚያ ጊዜያቸዉን የማጣራት ልምድ አሎት?

ሀ. አዎ ለ. የለኝም

1. ራስን በራስ በመድሃኒት ስለማከም ያለዎትን የግል አስተያየት ቢገልፁልን;

ሀ. እስማማለሁ

ለ. አልስማማም

ሐ. እንደ ህመሙ እና እንደ መድሀኒቱ ዓይነት

መ. የምለው የለኝም
